# Embryological Development and Topographic Anatomy of Pelvic Compartments—Surgical Relevance for Pelvic Lymphonodectomy

**DOI:** 10.3390/jcm10040708

**Published:** 2021-02-11

**Authors:** Andreas Bayer, Tillmann Heinze, Ibrahim Alkatout, Daniar Osmonov, Sigmar Stelzner, Thilo Wedel

**Affiliations:** 1 Kurt Semm Center for Minimally Invasive and Robotic Surgery, Center of Clinical Anatomy, Institute of Anatomy, Kiel University, 24098 Kiel, Germany; a.bayer@anat.uni-kiel.de (A.B.); t.heinze@anat.uni-kiel.de (T.H.); 2 Kurt Semm Center for Minimally Invasive and Robotic Surgery, Department of Obstetrics and Gynecology, Campus Kiel, University Hospital Schleswig Holstein, 24105 Kiel, Germany; 3 Kurt Semm Center for Minimally Invasive and Robotic Surgery, Department of Urology, Campus Kiel, University Hospital Schleswig Holstein, 24105 Kiel, Germany; Daniar.Osmonov@uksh.de; 4 Department of General Surgery, Hospital Dresden-Friedrichstadt, 01067 Dresden, Germany; Sigmar.Stelzner@klinikum-dresden.de

**Keywords:** pelvic compartments, embryologic development, oncologic surgery, pelvic lymphonodectomy, topographic anatomy, autonomic pelvic nerves, rectal cancer, uterine cancer, prostate cancer

## Abstract

Background: The oncological outcome of surgery for the treatment of pelvic malignancies can be improved by performing pelvic lymphonodectomy. However, the extent and regions of lymph node harvest are debated and require profound knowledge of anatomy in order to avoid collateral damage. Methods: The embryological development and topographic anatomy of pelvic compartments in relation to pelvic lymphonodectomy for rectal, uterine, and prostate cancer are reviewed. Based on pre-dissected anatomical specimens, lymph node regions and drainage routes of the posterior and urogenital pelvic compartments are described in both genders. Anatomical landmarks are highlighted to identify structures at risk of injury during pelvic lymphonodectomy. Results: The ontogenesis of urogenital and anorectal compartments and their lymphatic supply are key factors for adequate lymphonodectomy, and have led to compartment-based surgical resection strategies. However, pelvic lymphonodectomy bears the risk of injury to somatic and autonomic nerves, vessels, and organs, depending on the regions and extent of surgery. Conclusion: Embryologically defined, compartment-based resection of pelvic malignancies and their lymphatic drainage routes are based on clearly delineated anatomical landmarks, which permit template-oriented pelvic lymphonodectomy. Comprehensive knowledge of pelvic anatomy, the exchange of surgical concepts between specialties, and minimally invasive techniques will optimize pelvic lymphonodectomy and reduce complications.

## 1. Introduction

### 1.1. Lymphonodectomy

Surgery for the treatment of malignant disease is not limited to the affected organ alone. According to clinical guidelines across surgical specialties, surgery performed with a curative intention consistently involves the removal of lymph nodes along lymphatic drainage routes. Different types of lymphonodectomy (e.g., systematic, therapeutic, sentinel, sampling, debulking) have been described [1]. These are viewed as an integral part of the surgical procedure and the overall therapeutic concept for the underlying malignant disease. In radical surgery, the purpose of removing locoregional lymph node metastases is to improve the prognosis of disease. Moreover, lymphonodectomy allows exact postoperative staging of the underlying malignant disease and provides a basis for adjuvant therapy [2,3].

**Figure 1:**
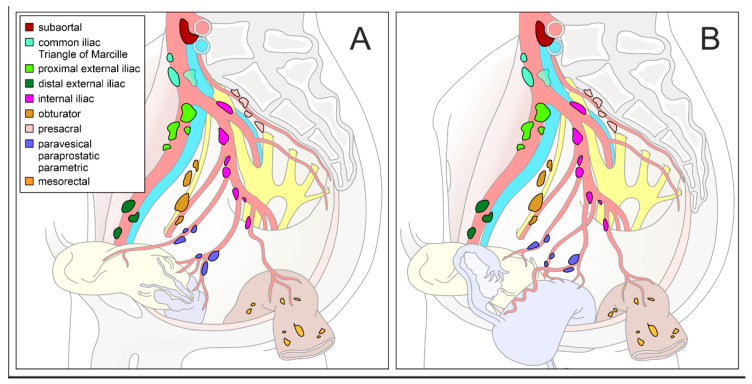
Schematic diagram of pelvic lymph node compartments. Mediolateral view of a right-sided male (**A**) and female (**B**) hemipelvis with pelvic organs and supplying arteries. Regional lymph nodes are colored differently (inserted legend in (**A**). Modified according to the Committee on Classification of Regional Lymph Nodes of the Japan Society of Clinical Oncology [4].

Lymphatic drainage of a given organ mainly occurs in centripetal direction and follows its blood supply which, in turn, is determined by the embryological development of the organ. Therefore, the extent and regions of lymphonodectomy must be based on the ontogenesis of the affected organ as well as the corresponding anatomical compartment. This concept is especially true of intrapelvic malignancies because pelvic organs have different embryological origins and are arranged in predefined compartments. Figure 1 provides a summary of major pelvic lymph node regions in the female and male pelvis.

### 1.2. Pelvic Compartments

The pelvic cavity is subdivided into two (male) or three (female) compartments. While the posterior compartment corresponds to the anorectum, the anterior compartment comprises the bladder and the prostate/seminal vesicles in males. In females, the additional middle compartment consists of the uterovaginal complex and the adnexa. Based on ontogenetic development, each of these compartments is marked by organ-specific lymphatic drainage routes, which have led to specific surgical approaches for lymphonodectomy.

Despite the diverse concepts and extent of pelvic lymphonodectomy propagated in colorectal, gynecologic, and urologic cancer surgery, similar technical challenges are faced in all of these surgical specialties. On the one hand, the removal of lymph nodes should be as radical as necessary. On the other hand, functional and structural damage should be as minimal as possible. A prerequisite for the achievement of these aims is profound knowledge of both, the ontogenetic and topographic anatomy of pelvic organs. These aspects will be addressed in the present work by briefly recapitulating embryologic origins, describing the anatomical features of each pelvic compartment, and surgical concepts derived from these. Anatomical landmarks that must be preserved during pelvic lymphonodectomy are given special attention. Although the anatomical features apply to all types of surgery (open, laparoscopic, robot-assisted approaches), they are particularly important for minimally invasive techniques because the surgeon’s topographic orientation must be aligned to the limited and optically magnified surgical fields.

## 2. Methods

### 2.1. Selection of Pelvic Malignancies

The most common malignancies in each pelvic compartment were selected to illustrate radical surgical approaches and the respective concepts of lymphonodectomy. Rectal cancer was chosen for the posterior compartment, cervical/uterine cancer for the middle compartment, and prostate cancer for the anterior compartment. Although the surgical regimen for these cancers is organ specific, pelvic lymphonodectomy is performed within anatomical regions of overlapping interdisciplinary interest. A comprehensive description of pelvic topographic anatomy appears to be mandatory for all involved surgical specialties (colorectal, gynecological, urological surgery).

### 2.2. Dissection of Anatomical Specimens

Body donors were recruited from the body donation program at the Institute of Anatomy, Christian-Albrechts University of Kiel, after previous written consent had been obtained for educational and research purposes. After formalin (3%) perfusion fixation via femoral arteries and subsequent fixation in ethanol (70%), pelvic specimens were removed and sectioned either transversely or sagittally for macroscopic dissection. Pelvic lymph node regions, pelvic organs with their respective blood vessels, and anatomical structures at risk during lymphonodectomy were exposed for each pelvic compartment. In selected cases, regional lymph nodes were first identified and then removed in a stepwise manner to simulate an extended pelvic lymphonodectomy and demonstrate the anatomical topography before and after lymph node dissection. Two female (67 and 70 years old) and three male (65, 75, and 81 years old) pelvic specimens with no evidence of pelvic disease or previous surgery were used for photographic illustration. Photographs were taken with a digital camera (Sony Alpha 7.III, 35 mm full frame with Sony FE 90 mm F2.8 Macro GOSS lens, Sony Corporation, Tokyo, Japan) and processed with compatible software (Sony Remote Version 1.4.00.01241, Sony Corporation, Tokyo, Japan; Adobe Photoshop CS6 2012, San Jose, California, USA. Structures of interest were highlighted with different semitransparent colors using the CorelDRAW software (Version 2019, Ottawa, ON, Canada).

## 3. Posterior Pelvic Compartment

### 3.1. Embryology

The primitive gut is an endoderm-derived organ system subdivided into the foregut, midgut and hindgut, supplied by the celiac trunk, the superior and the inferior mesenteric arteries, respectively. Derivatives of the hindgut comprise the left colic flexure, the descending and sigmoid colon, the rectum, and the upper anal canal. During early embryologic development, the anorectal tube is still connected to the urogenital system via the endodermal cloaca, resembling a common pouch closed in the caudal aspect by the cloacal membrane. An emerging urorectal septum subdivides the cloacal cavity into a ventrally located urogenital sinus and a dorsally located anorectal canal. Once the cloacal membrane vanishes, the anal canal is temporarily closed by the anal membrane. At embryonic week 9, the anal canal reopens at the level of the dentate line, connecting the upper endoderm-derived anal canal with the lower ectoderm-derived one [5]. Given the endodermal origin of the rectum, this last segment of the hindgut is mainly supplied by the superior rectal artery, which is a branch of the inferior mesenteric artery. Thus, blood supply as well as lymphatic routes of the rectum are located in perirectal tissue, also referred to as the mesorectum.

### 3.2. Surgery

Translation of these embryological considerations into surgical concepts for rectal cancer was achieved by the introduction of total mesorectal excision (TME) [6], which was implemented and promoted by Richard Heald [7]. The concept of TME takes the embryological origin of the rectum into account by completely removing the mesorectal tissue as an intact package harboring the lymphatic drainage of this organ. Dissection is performed along an embryologically defined avascular plane (“holy plane”) between the mesorectal and parietal pelvic fascia, thus allowing complete harvest of mesorectal lymph nodes as well as preservation of pelvic autonomic nerves. TME can be performed either transabdominally by laparotomy, laparoscopy, robot-assisted surgery [8,9,10], or by transanal approach [11].

While TME addresses the main lymphatic route of the rectum, the distal anorectal segment is additionally drained by the internal iliac route via the middle rectal and pudendal vessels. The frequency of the middle rectal artery is reported to range from 12% to 97%. Therefore, its relevance for lymphatic drainage is not fully understood [12]. Progression of advanced rectal cancer along the lateral rectal pedicles may lead to so-called lateral spread of the disease, affecting lymph nodes of the pelvic sidewall. However, the need for extended lateral pelvic lymphonodectomy in addition to TME for curative treatment of primary rectal cancer is still under debate. Currently it is agreed that pre-existing enlarged lymph nodes must be addressed, either by radiotherapy or surgery [13]. Recent data indicate that acceptable rates of disease-free and overall survival can be achieved by neoadjuvant chemoradiotherapy with selective lateral pelvic lymph node dissection [14].

### 3.3. Anatomy

**Figure 2:**
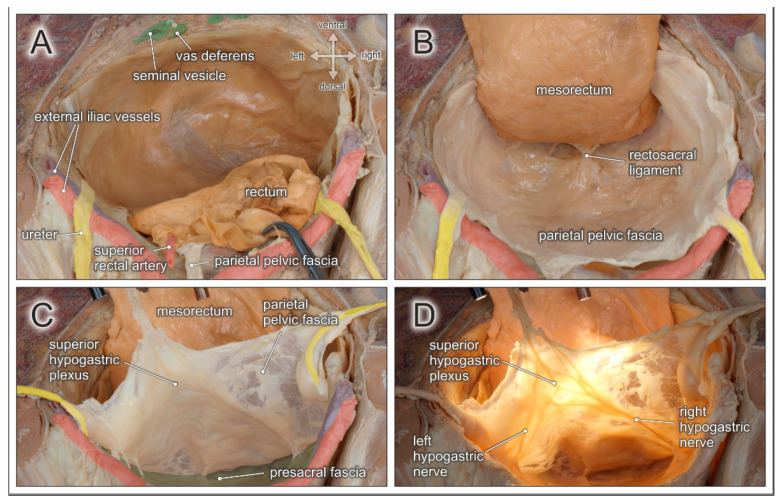
Topographic anatomy of the posterior pelvic compartment. Dorsocranial view of a male pelvis. (**A**) The rectum and the mesorectum with the superior rectal artery are transected at the rectosigmoid junction (clamp). (**B**) Dorsolateral mobilization of the mesorectum in a TME-like manner between the parietal pelvic fascia and the mesorectal fascia along the retrorectal space. Both fasciae are fused by the rectosacral ligament. (**C**) The parietal pelvic fascia and both ureters are lifted to expose the presacral space behind the presacral fascia. (**D**) Diaphanoscopy of the parietal pelvic fascia reveals the embedded superior hypogastric plexus and both hypogastric nerves.

The mesenteries are responsible for blood supply and lymphatic drainage of the entire gastrointestinal tract. The mesorectum corresponds to the most caudal part and is composed of perirectal adipose tissue, harboring branches of the superior rectal artery and the mesorectal lymph nodes (see Figure 2A). Mesorectal tissue is most developed at its dorsolateral aspect (so-called mesorectal cheeks), becomes thinner along the ventral rectal wall, and is circumferentially enveloped by the visceral pelvic fascia (mesorectal fascia). While the mesorectal fascia is contiguous in its dorsal and ventral aspect, it is pierced bilaterally by rectal nerves originating from the inferior hypogastric plexus and small branches of middle rectal arteries (if present). These connections between the mesorectum and the pelvic sidewall correspond to the paraproctium, frequently referred to as the lateral rectal ligaments, rectal pedicles, or T-junctions. Subsequently, complete surgical mobilization of the mesorectum requires sharp dissection laterally, while posterior and anterior mesorectal dissection can be achieved by using “self-opening” surgical planes. The correct surgical plane for TME corresponds to an avascular interface between the mesorectum and the parietal pelvic fascia, characterized by loose areolar connective tissue (also known as angel’s hair) (see Figure 2B). Dissection in this embryologically determined retrorectal space [15], resembling the “innermost dissectable perirectal layer” (personal communication from Richard Heald), provides the basis for complete removal of an intact lymphovascular mesorectal package.

The parietal pelvic fascia covers the inner surface of the pelvic wall. Due to its bilaminar structure, the fascia envelopes the pelvic autonomic nerve plexus and the ureters (see Figure 2C). Dorsally, the parietal pelvic fascia is adjacent to the presacral fascia, which covers the medial and lateral sacral arteries and the presacral venous plexus running along the sacral concavity. The presacral space is located between the parietal pelvic fascia and the presacral fascia. This interface has similar morphological features as the retrorectal space (self-opening plane with loosely arranged connective tissue), and may therefore be easily mistaken for the proper dissection plane for TME. However, following this plane would result in the excision of pelvic autonomic nerves coursing within the parietal pelvic fascia and lead to autonomic denervation of pelvic organs. Approximately at the fourth sacral vertebra, all fascial layers fuse in the midline and are densely connected to the posterior rectal wall via the rectosacral ligament [16] (see Figure 2B).

**Figure 3:**
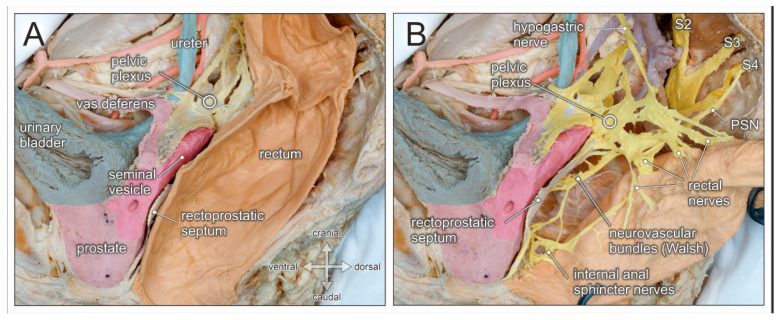
Topographic anatomy of the posterior pelvic compartment. Medial view of a right-sided male hemipelvis. The parietal pelvic fascia is removed to visualize the embedded autonomic pelvic nerves. (**A**) The posterior pelvic compartment is delimited from the urogenital compartment by the rectoprostatic septum (Denonvilliers fascia). (**B**) The rectum is pulled aside to reveal the inferior hypogastric/pelvic plexus, the hypogastric nerve, and the pelvic splanchnic nerves (PSN, from sacral nerve S4). The inferior hypogastric nerve gives rise to rectal nerves and more caudally to internal anal sphincter nerves, as well as the neurovascular bundle of Walsh.

Preservation of the autonomic pelvic nerves which govern anorectal and urogenital functions is best achieved by respecting the parietal pelvic fascia. In fact, the superior hypogastric plexus, the hypogastric nerves, and the inferior hypogastric plexus are all ensheathed in this bilaminar pelvic fascia (see Figure 2D). The elaborate network of the inferior hypogastric plexus is fed by sympathetic input from the hypogastric nerves, and by parasympathetic input from pelvic splanchnic nerves originating from the second, third, and fourth ventral sacral nerves (see Figure 3B). At the level of the rectal pedicles, rectal branches diverge from the main nerve plexus and enter the mesorectum. The inferior hypogastric plexus continues ventrally to supply the seminal vesicles, distal ureters, bladder, and vasa deferentia. More caudally, nerve fibers extend towards the prostatic apex and supply the internal urinary sphincter and the cavernous bodies (the neurovascular bundle of Walsh). Nerve fibers also approach the anterolateral aspect of the anorectal junction and supply the internal anal sphincter [17]. The ventral margin of the posterior compartment is delineated by the rectoprostatic septum in males and the rectovaginal septum in females (see Figure 3A). Anterolaterally, the neurovascular bundles are closely related to the rectogenital septum. However, as most autonomic nerves responsible for the mediation of urogenital functions extend in front of the rectogenital septum, dissection behind this septum is the preferred approach for nerve-preserving anterior mobilization of the rectum.

In advanced low rectal carcinoma, lateral lymphatic spread towards the pelvic sidewall via the lateral rectal pedicles along the middle rectal arteries must be taken into account. In these cases, lymphonodectomy will address those regions of the lateral pelvic wall that harbor suspicious lymph nodes. These regions include lymphatic tissue surrounding the common and internal iliac vessels and the obturator fossa. During lymphonodectomy, the parietal pelvic fascia ensheathing the inferior hypogastric plexus should be respected as the medial border of the dissection plane, unless the tumor has spread into these structures. While the obturator nerve must be preserved, the obturator vessels may be removed [13].

## 4. Middle (Female) Pelvic Compartment

### 4.1. Embryology

The female reproductive tract is derived from three different primordial tissue complexes: cranially from the Müllerian tubercle complex, in the middle from the deep urogenital sinus and the vaginal plate complex, and caudally from the superficial urogenital sinus genital folds and the tubercle complex [18]. The Müllerian tubercle complex is the origin of the uterine tubes, the uterus, and the upper part of the vagina. During early embryologic development, the Müllerian ducts develop bilaterally along the urogenital crests from the mesoderm, as elongated indentations of the coelomic epithelium. The Müllerian ducts run parallel to the urinary tract, opening distally into the upper urogenital sinus. During male embryological development, the Müllerian ducts regress due to the anti-Müllerian hormone produced in Sertoli cells of the fetal testicles. As female fetuses lack Sertoli cells and are unable to produce anti-Müllerian hormone, the Müllerian ducts further differentiate under the influence of estrogens to give rise to the major components of internal female genital organs. In fact, the bilaterally located Müllerian ducts develop into the uterine tubes on both sides. Along the midline both Müllerian ducts fuse to give rise to the uterus. If the fusion of the Müllerian ducts is incomplete, a bicornuate uterus may develop. Moreover, the entire uterine cervix (supravaginal and vaginal portion) as well as the upper third of the vagina originate from the Müllerian ducts. Thus, the uterine tubes, uterus/cervix and upper vagina are all derivates of the paramesonephric ducts and resemble the so-called Müllerian compartment. The Müllerian compartment is mainly supplied by uterine and vaginal blood vessels originating from internal iliac vessels and drained by lymphatic vessels extending via the mesometrium towards the pelvic side wall.

### 4.2. Surgery

As the uterine cervix belongs to the Müllerian compartment, which is primarily connected to mesometrial lymph nodes and their corresponding drainage routes along the iliac vessels, total mesometrial resection (TMMR) has been proposed as the surgical approach for curative resection of cervical cancer [19]. As this concept adheres to, and fits best, the embryological considerations outlined above, it is the main focus of this anatomy-based report. Radical hysterectomy in accordance with the principles of TMMR involves excision of the derivatives of the Müllerian ducts, including the vascular and ligamentous mesometrium, followed by therapeutic lymphonodectomy. TMMR can be performed by open, laparoscopic, or robot-assisted techniques [20,21]. However, long-term data on oncologic survival are available so far only for the open approach, while data confirming non-inferiority for minimally invasive approaches are still lacking.

Analogous to the concept of TME introduced for rectal cancer surgery, TMMR is based on an ontogenetic, compartment-based resection template corresponding to the Müllerian morphogenetic unit, which is permissive for malignant propagation and progression [22]. The results of a prospective observational single-center cohort study revealed good local tumor control and good survival outcomes in patients with cervical cancer treated with TMMR guided by stage-associated ontogenetic cancer fields and removal of associated lymph nodes without adjuvant radiotherapy [23]. On the one hand, TMMR is aimed at radical removal of the complete Müllerian compartment. On the other hand, extra-compartmental organs of different embryonic origins (e.g., ureters, urinary bladder, rectum, autonomic pelvic nerves) can be fully preserved despite their close vicinity to the tumor because compartment margins remain intact and undisrupted.

Therapeutic lymphonodectomy during TMMR includes removal of mesometrial, paravisceral, external iliac, common iliac, and presacral lymph nodes, defined as first-line lymph nodes, depending on the pattern of lymphatic spread [24]. In case of diseased first-line lymph nodes, those further downstream are resected additionally. The latter are referred to as second- and third-line lymph nodes. Depending on the stage of cervical cancer and the pattern of regional metastases, the regions include inframesenteric, infra- and suprarenal, periaortic, and pericaval lymph nodes [23]. The relevance of these surgical fields holds true for cervical as well as endometrial cancer.

### 4.3. Anatomy

**Figure 4:**
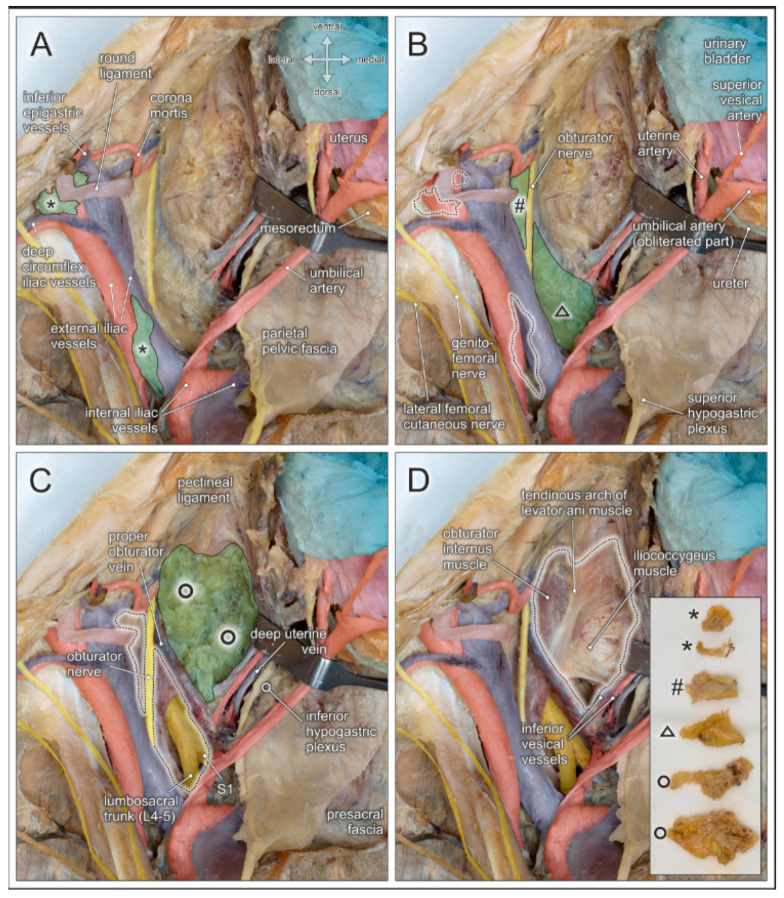
Topographic anatomy and stepwise pelvic lymphonodectomy. Dorsocranial view of a left female pelvis. Pelvic organs (urinary bladder, uterus, rectum, ureter) and their blood vessels are shifted to the right side (hook), and the round uterine ligament is transected. Regional lymph nodes are highlighted in green and dotted black lines after removal. (**A**) Distal and proximal external iliac lymph nodes (asterisks) are located in the vicinity of the deep circumflex iliac vessels, the corona mortis, and the genitofemoral nerve. (**B**) Lymph nodes of the obturator fossa (hashtag) are closely related to the obturator nerve and vessels, and the corona mortis. Presacral lymph nodes (triangle) extend along the sacral concavity and cover the sacral spinal nerves and the lumbosacral trunk (depicted in C after removal). (**C**) Paravisceral/paravesical lymph nodes (circles) are located between the pelvic sidewall and the urinary bladder, extending along the surface of the levator ani muscle. (**D**) Complete removal of pelvic lymph node regions with exposure of relevant anatomical structures at risk of potential injury during pelvic lymphonodectomy. Insert shows the lymphatic tissue harvested from the regions indicated in (**A**–**C**).

Given the several intrapelvic regions to be addressed during lymphonodectomy when performing radical hysterectomy for cervical cancer, detailed knowledge of the corresponding topographic anatomy is mandatory. Figure 4 illustrates stepwise removal of the relevant lymph node compartments and highlights the anatomical structures at potential risk of injury.

**Figure 5:**
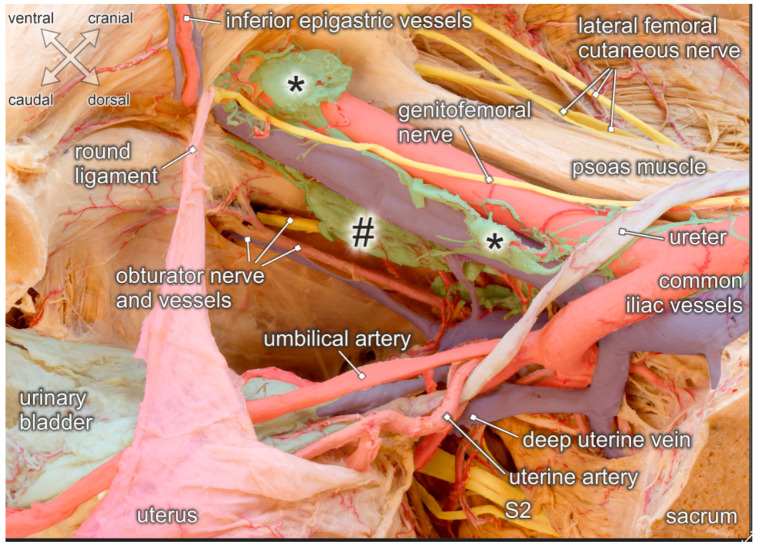
Topographic anatomy of the pelvic sidewall. Medial view of a right female hemipelvis. The bladder and uterus are shifted to the left side; fascia and parametric tissue are removed. Distal and proximal external iliac lymph nodes (asterisks), obturatory lymph nodes (hashtag) and interconnecting lymphatic vessels are exposed. Structures at risk during lymphonodectomy are the genitofemoral nerve running across the external iliac vessels, the obturator nerve and vessels extending throughout the obturator fossa, and the ureter crossing the proximal external iliac vessels. While both, the umbilical and uterine artery are undercrossed by the ureter, the deep uterine vein runs beneath the ureter.

External iliac lymph nodes in the distal aspect are located next to the deep inguinal ring, in close proximity to the branches of the genitofemoral nerve, and are crossed by the deep circumflex iliac vessels (see Figure 4A and Figure 5). Neural structures, and especially the deep circumflex iliac vein, are endangered during removal of these lymph nodes. External iliac lymph nodes in the proximal aspect are located along, or intercalated between, the external iliac artery and vein. These lymph nodes are flanked laterally by the genitofemoral nerve which passes upon the psoas muscle. At the level of the iliac bifurcation, the ureter crosses the external iliac vessels and can be easily injured because of its superficial course. Common iliac lymph nodes extend on both sides of the common iliac vessels to the aortic bifurcation. Endangered nerves during lymph node removal include the obturator nerve running laterally along the border of the psoas muscle and the lumbosacral trunk (L4–L5), in the medial aspect, adjacent to the presacral region.

Lymph nodes within the obturator fossa extend mediocaudally to the external iliac vein, covering the internal obturator muscle and the tendinous arch of the levator ani muscle (see Figure 4B and Figure 5). The main structures at risk are the obturator nerve and, more caudally, the obturator vessels passing towards the inner opening of the obturator canal below the superior pubic ramus. Lymphatic tissue can be found in the superior and inferior aspect of the obturator nerve. Therefore, complete removal requires clear identification and preservation of this nerve. Particular attention must be given to anastomotic branches between the obturator vessels and the external iliac/inferior epigastric vessels, also known as corona mortis. The frequencies of arterial and venous corona mortis are reported to be 8–65% and 17–60%, respectively, depending on the pattern of branching and anastomosis [25].

The paravisceral lymph node compartment in women corresponds to the paravesical tissue extending between the pelvic sidewall and the urinary bladder (see Figure 4C). Complete removal of lymphatic tissue exposes the surface of the levator ani muscle and its tendinous arch originating from a condensation of the obturator fascia (See Figure 4D). Inferior vesical vessels branching from the internal iliac artery and reaching the bladder neck must be preserved.

Presacral lymph nodes extend along the sacral concavity, and are located in the vicinity of branches from the posterior division of the internal iliac vessels, such as the superior gluteal and lateral sacral vessels. During removal of these lymph nodes, ventral spinal nerves (mainly S1–S2) departing from the sacral foramina and the lumbosacral nerve trunk (L4–L5), and descending over the pelvic brim to join the first sacral nerve are endangered.

According to Cibula et al. [1], these pelvic lymph node regions are interconnected and drained by two major lymphatic trunks coursing along the pelvic sidewalls. While a superficial trunk passes ventrally to the external and common iliac vessels and continues to the precaval/preaortic regions, a deep trunk courses more medially, crosses the obturator fossa, and divides into two segments which flank the common iliac vessels on either side. After collecting lymph nodes from the presacral and internal iliac regions, the deep trunk enters the pre/paraaortic lymph node basin.

Despite its radical nature, lymphonodectomy should not compromise the autonomic pelvic nerves because the latter are essential for the preservation of anorectal and urogenital functions. In the TME procedure for the posterior pelvic compartment as well as in radical hysterectomy involving the middle pelvic compartment, care should be taken to ensure that the inferior hypogastric plexus embedded within the parietal pelvic fascia and the pelvic splanchnic nerves are preserved. Thus, nerve-sparing radical hysterectomy is aimed at the transection of only those nerves that branch off from the inferior hypogastric plexus via the vascular mesometrial/paracervical tissue into the uterus, while nerves supplying the rectum, and especially the bladder, are preserved [26].

## 5. Ventral (Male) Pelvic Compartment

### 5.1. Embryology

Differentiation of the Müllerian ducts (paramesonephric ducts) is further promoted by the absence of the anti-Müllerian hormone in female embryos. In male embryonic development, the paramesonephric components degenerate and the Wolffian ducts (or mesonephric ducts) give rise to the male urogenital organs. The Wolffian ducts are connected to the urogenital sinus and form the epididymis, the vas deferens, seminal vesicles, and the trigone of the urinary bladder. The remaining bladder components, the prostate and the urethra develop from the urogenital sinus. Initially, both pelvic compartments—the posterior rectal and the anterior urogenital compartment—are connected by opening together into the primitive urogenital sinus (common cloaca). However, the two organ systems are subsequently separated by an ingrowing urorectal septum subdividing the urogenital sinus into a ventral portion connected to the Wolffian duct and a dorsal portion giving rise to the anorectal canal. Thus, at the end of embryologic development, the distal connection between the two pelvic compartments is completely detached and separated by the rectoprostatic septum (Denonvilliers fascia).

### 5.2. Surgery

Surgical treatment of both localized and locally advanced prostate cancer with curative intention consists of radical prostatectomy and lymphonodectomy. While the organ-related technical procedures of radical prostatectomy are well defined and standardized, the extent of pelvic lymphonodectomy is still a debated issue [27,28]. Standard lymphonodectomy is limited to the removal of lymph nodes in the obturator fossa and along the external iliac vessels, and has been recommended for locally limited prostate cancer in patients with a low risk profile. However, patients with locally advanced prostate cancer and/or a high risk profile may undergo extended pelvic lymphonodectomy combined with radical prostatectomy [29]. Extended template-based pelvic lymphonodectomy includes the removal of lymph nodes lining the internal and common iliac vessels, as well as presacral lymph nodes. A sufficient number of lymph nodes must be harvested for an optimal clinical outcome [27]. In cases of recurrent prostate cancer, some authors recommend a salvage extended pelvic lymph node dissection, which includes the additional removal of interiliac/subaortic and paraaortic lymph nodes [30]. Marcille’s triangle or fossa has been given special attention in the creation of adequate anatomical templates for lymph node dissection. Lymph nodes located in this region are covered by the iliac vessels and therefore less obvious, but are considered relevant for achieving optimal lymph node clearance [31,32,33].

### 5.3. Anatomy

The functional outcome of radical prostatectomy must include the preservation of pelvic autonomic nerves because they govern urinary and fecal continence as well as sexual functions. Accordingly, nerve-sparing radical prostatectomy introduced by Patrick C. Walsh and based on anatomical dissection studies performed together with Pieter J. Donker (1981) has become the standardized surgical procedure for prostate cancer [34]. Similar to the TME procedure for rectal cancer in the posterior pelvic compartment and radical hysterectomy in the middle pelvic compartment, nerve-sparing techniques in the anterior pelvic compartment are aimed at removal of the affected organs while ensuring the integrity of the autonomic pelvic nerves. This is particularly true of the neurovascular bundles (also known as the bundle of Walsh) which originate from the caudal portion of the inferior hypogastric plexus, descend along the rectoprostatic septum, extend towards the prostatic apex and the urethral sphincter complex, and finally enter the penile cavernous bodies (see Figure 3).

**Figure 6:**
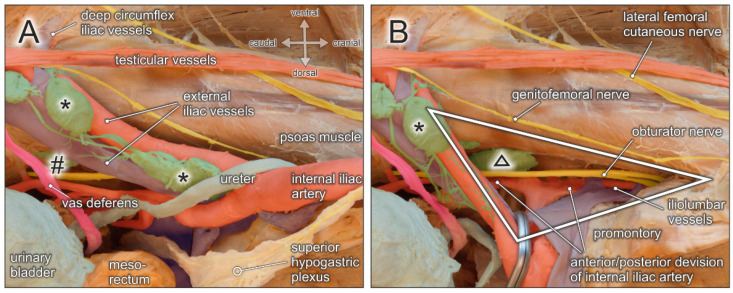
Topographic anatomy of the pelvic sidewall. Medial view of a right male hemipelvis. The urinary bladder and the mesorectum/rectum are shifted to the left side. (**A**) External iliac lymph nodes (asterisks), obturatory lymph nodes (hashtag), and interconnecting lymphatic vessels are exposed. Structures at risk during lymphonodectomy are the genitofemoral nerve running lateral along the external iliac artery, testicular vessels and the vas deferens, the obturator nerve and vessels extending throughout the obturator fossa, the ureter crossing the proximal external iliac vessels, and the hypogastric nerve. (**B**) External iliac vessels (clamp) and the ureter are shifted medially to the contralateral/left side to expose the triangle of Marcille (white triangle) limited by the fifth lumbar vertebra/promontory, the medial border of the psoas muscle, and the lateral aspect of the sacral concavity. Structures at risk during removal of lymph nodes (black triangle) within the triangle of Marcille are the proximal segment of the obturator nerve, the lumbosacral trunk (L4–L5), and the anterior and posterior division of the internal iliac vessels, in particular the iliolumbar vessels.

In lymphonodectomy for prostate cancer, the crucial anatomical landmarks are quite similar to those described for lymphonodectomy in cervical cancer. In both procedures, the lymphatic drainage routes along the laterodorsal pelvic sidewall must be addressed. Thus, structures at risk include the deep circumflex iliac vein crossing the distal external iliac artery, the genitofemoral nerve running along the medial border of the psoas muscle, the obturator nerve and vessels within the obturator fossa, and the ureter crossing the iliac bifurcation(see Figure 6A). In extended lymphonodectomy, which additionally includes lymph nodes lining the common iliac vessels and the presacral region, attention should be given to the ureter at the pelvic brim and, more medially, to the superior hypogastric plexus and hypogastric nerves embedded within the parietal pelvic fascia (Figure 6).

A rather neglected lymph node region extends behind the proximal iliac vessels and corresponds to the triangle or fossa of Marcille, which is explored surgically by some surgeons during extended or salvage lymphonodectomy [31,32,33]. Marcille’s triangle is limited by the anterolateral aspect of the fifth lumbar vertebra, the medial border of the psoas muscle, and the ala of the sacrum (see Figure 6B). The base of the triangle projects onto the transverse process of the fifth lumbar vertebra and the lumbosacral and iliolumbar ligaments, which extend across the ala of the sacrum to the sacroiliac joint [35]. Access to Marcille’s triangle can only be achieved by full exposure, mobilization, and medial retraction of the external iliac vessels together with the ureter (Figure 6). The obturator nerve crosses this region laterocranially, followed by the lumbosacral trunk (L4–L5) more mediocaudally. Moreover, branches from the posterior division of the internal iliac vessels are exposed. These include iliolumbar vessels running in cranial direction, lateral sacral vessels extending towards the sacral concavity, and superior gluteal vessels descending into the suprapiriform foramen.

## 6. Lymph Nodes and Lymphatic Vessels

**Figure 7:**
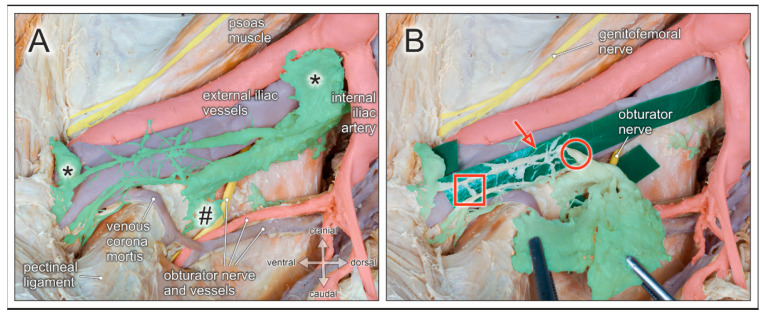
Lymph nodes and lymphatic vessels of the pelvic sidewall. Medial view of a right male hemipelvis. Pelvic organs, their blood vessels and autonomic nerves are shifted to the contralateral side; fatty tissue is removed. (**A**) External iliac lymph nodes (asterisks), obturator lymph nodes (hashtag), and interconnecting lymphatic vessels are highlighted (green). The obturator nerve is partly concealed by lymphatic tissue in the obturator fossa. Venous corona mortis is discernible. (**B**) The lymphatic tissue is partly pulled out of the obturator fossa en bloc (forceps). Green plastic strips are inserted to expose the morphological features of lymphatic vessels arranged in finely meshed networks extending between lymph nodes. Lymphatic vessels are of small (red arrow) and large (red circle) diameters, with rope ladder-like ramifications (red square), and susceptible to mechanical damage due to their thin walls.

The commonly used terms *lymphonodectomy*, *lymphadenectomy* or *lymph node dissection* suggest that only lymph nodes are dissected and removed. However, surgical clearance of lymphatic drainage routes includes concomitant harvesting of lymphatic vessels/trunks that connect the different lymph node regions. Lymphatic fluid enters into a lymph node via several afferent lymphatic vessels, passes through the cortex and medulla, and is drained into efferent lymphatic vessels at the lymph node hilum to reach the next collecting lymph node station. Thus, lymphatic drainage routes resemble a finely meshed network of lymphatic vessels with intercalated lymph nodes. These morphological features become particularly obvious when the surrounding fatty tissue is meticulously removed to expose the lymphatic vascular network (Figure 5, Figure 6 and Figure 7).

Whereas lymph nodes possess a rather robust fibrous capsule, lymphatic vessels have thin walls and an inner endothelial lining only surrounded by a thin muscular and adventitial layer. Moreover, lymphatic tissue is embedded within fatty and connective tissue, has a pale appearance, and is therefore not easily discernible. Given these peculiarities, lymphatic vessels are easily prone to injury due to mechanical or thermal factors during surgical lymphonodectomy. Rupture of afferent as well as efferent lymphatic vessels is more likely to occur when lymph nodes are harvested by blunt dissection or gross plucking (Figure 7). Complications resulting from surgically induced injury of lymphatic vessels during pelvic lymphonodectomy include lymphatic fistula, lymphocele, chylopelvic fistula, chylous ascites, and subsequent wound infection [36,37]. These complications can be reduced by skilled and careful manipulation of lymphatic tissue, en bloc harvesting, and adequate sealing of lymphatic vessels.

## 7. Discussion

The removal of malignant tumors with large safety margins has been the traditional approach in surgical oncology. This has been replaced by embryologically defined compartment-based surgery for several cancer entities. According to this concept, malignant tumors and their lymphatic drainage routes are resected in the anatomical compartments derived from their embryological differentiation [38]. This approach is supported by insights into the early embryologic development of the lymphatic system consisting of lymphatic primordia which give rise to several lymphatic sacs/basins (e.g., bilateral iliac lymphatic plexus) [39]. These lymphatic regions and therein developing lymph nodes are specific for each region and allow selective surgical removal. Therefore, structures and/or organs, although anatomically located in close proximity to the tumor, may be left in situ without worsening the prognosis of disease, because they are derived from a different embryological compartment, drained by different lymph node basins, and thus not primarily involved in the progression of malignancy. In fact, surgical procedures following this concept could not only improve oncological outcomes, but should also reduce operation-related morbidity by preserving functionally relevant structures outside the affected compartment [19].

As for pelvic malignancies, the above-mentioned concept was first applied to rectal carcinoma by introducing TME [7]. TME involves the removal of the rectal tumor with its main lymphovascular tissue enclosed by the mesorectum, while preserving pelvic autonomic nerves for the maintenance of urogenital functions. This surgical technique has considerably improved the prognosis of disease and been adopted worldwide as the standard surgical approach for rectal cancer [6]. In contrast, the benefit of additional “prophylactic or indicated” lateral lymph node dissection is still a debated issue [13]. This procedure is mainly performed in the eastern hemisphere, while neoadjuvant treatment regimens followed by TME are favored elsewhere. Recent studies show that a combination of these approaches could further improve the prognosis of disease, especially in patients with advanced disease [40]. In view of longer operating times and the risk of complications, lateral lymph node dissection could be omitted in patients with low-stage tumors [41]. If a lymphonodectomy is performed at the pelvic sidewall in patients with rectal cancer, colorectal surgery will face the same technical challenges and should adhere to the same anatomical landmarks as those encountered in pelvic lymphonodectomy for urogenital malignancies.

Analogous to the TME procedure in rectal cancer surgery, total mesometrial excision (TMMR) was introduced in surgery for cervical cancer by Michael Höckel [19,42]. TMMR is based on the fact that the uterine tubes, the uterus, and the cranial vagina develop from the Müllerian ducts. TMMR for cervical cancer includes the removal of paravisceral, external and common iliac, and presacral lymph nodes [43] and is based on the pioneering work of Günther Reiffenstuhl, who described the lymphatic system of the female genital organs and its surgical relevance for cervical/uterine cancer [44]. Given the prognostic importance of lymph node status [45], additional resection of the inframesenteric, infrarenal and suprarenal periaortic/pericaval lymph nodes may also be indicated, depending on the tumor stage [43]. In line with the improved prognosis of rectal cancer by the use of TME, this embryologically defined compartment-based surgical procedure has improved local tumor control and the overall oncologic prognosis in cervical cancer [19,22,23]. Although not widely accepted or generally performed, extended mesometrial resection based on the ontogenetic concept can also be used in locally advanced or relapsed cervical cancer [46,47].

A variety of pelvic lymphonodectomy procedures have been proposed for cervical cancer, depending on the extent of lymph node dissection [1]. Whereas type I dissection is limited to the superficial external and common iliac lymph nodes and obturator lymph nodes above the obturator nerve, type II and type III dissection involves the extension of lymphonodectomy to the deep external, internal, common iliac, caudal obturator lymph nodes and the presacral region, including exposure of the lumbosacral trunk.

In radical prostatectomy for prostate cancer, the standard approach of lymphonodectomy has traditionally been limited to the area of the obturator fossa and the external iliac vessels. Although still controversially discussed [48,49], many centers recommend extended lymphonodectomy in accordance with the embryologic origins and lymphatic drainage routes of the ventral pelvic compartment when performing primary curative resection [29]. This involves the removal of lymph nodes along the common and internal iliac vessels as well as in the presacral region and the triangle of Marcille [31]. Extensive pelvic lymphonodectomy has been shown to improve the prognosis of disease, especially in patients with intermediate- or high-risk prostate cancer [50]. Moreover, the additional removal of interiliac and paraaortic lymph nodes has been advised in patients with local recurrence [30].

Despite the proven prognostic relevance of lymphonodectomy for pelvic malignancies in all three pelvic compartments, the anatomical terminology and topographic delineation of pelvic lymph node basins are not uniformly defined across surgical disciplines. For example, the terms *interiliac*, *subaortic*, *paravisceral*, *presacral* or *internal iliac* lymph node regions are interpreted differently by surgeons of various specialties, such as urology, gynecology, or surgery. These diversities may give rise to biased data concerning the description, extent, and potential benefits of lymphonodectomy in patients with pelvic malignancies. To overcome this drawback, pelvic lymph node regions should be defined as precisely as possible by clear delineation of their topographic boundaries. This will provide reliable anatomical landmarks for orientation during dissection. A standardized classification and terminology of lymph node regions will optimize interdisciplinary and international research related to lymphonodectomy procedures. The Committee on Classification of Regional Lymph Nodes of the Japan Society of Clinical Oncology has published guidelines which have addressed these requirements and should be used in future studies [4].

Current surgical approaches are rapidly shifting from open to laparoscopic and robot-assisted procedures. On the one hand, these minimally invasive approaches are technically more demanding because of the limited operating field, unfamiliar surgical access routes, and hampered anatomical orientation. On the other hand, minimally invasive techniques and especially robot-assisted interventions offer optimal degrees of freedom for surgical instruments, three-dimensional and magnified visualization, and tremor-free manipulation of anatomical structures. These advantages permit the surgeon to overcome the challenges of pelvic lymphonodectomy, which include precise removal of lymphatic tissue and meticulous preservation of adjoining susceptible anatomical structures [10,21,51,52]. The increasing acceptance of minimally invasive robot-assisted techniques for pelvic lymphonodectomy will further improve oncological outcomes as well as the functional integrity of pelvic organs. However, regardless of the surgical approach, profound knowledge of the anatomy of pelvic compartments will remain an essential prerequisite for successful radical surgery in patients with pelvic malignancies.

## Data Availability

All data supporting the reported results are archived in the Institute of Anatomy at Kiel University. Publicly archived datasets were not analyzed ot generated during the study.
